# Prediction of pavement water film depth and estimation of critical rainfall conditions for refined road safety management: A simulation study

**DOI:** 10.1371/journal.pone.0318228

**Published:** 2025-02-13

**Authors:** Jinliang Xu, Wenzhen Lv, Chao Gao, Tian Xin, Xiantong Liu, Yahao Hou

**Affiliations:** School of Highway, Chang’an University, Xi’an, Shaanxi, China; Shandong University of Technology, CHINA

## Abstract

The development of a smart expressway ensuring all-weather safe access represents the future trajectory of transportation infrastructure. A key task in this advancement is the precise prediction of water film depth (WFD) on road surfaces. Conventional WFD prediction models often assume constant grade and cross slope, an oversimplification that may affect predictive accuracy. In this study, typical highway alignments were meticulously modeled in three dimensions (3D) using Building Information Modeling (BIM) technology, and WFD simulations were conducted using a coupled discrete phase model and Eulerian wall film model (DE-WFD model). Simulation results revealed that the DE-WFD model consistently predicts higher WFD compared to the RRL and PAVDRN models. In contrast, its predictions are approximately 0.12 mm (40%) lower than those of the Gallaway model when rainfall intensity is below 7.8 mm/h. At higher rainfall intensities, DE-WFD predictions closely align with the Gallaway model. Field tests conducted with a feeler gauge of 0.01 mm resolution confirmed the accuracy of these predictions, showing a maximum deviation of just 7% between predicted and measured values. Additionally, the study assessed the sensitivity of the DE-WFD model to variations in grade and cross slope along the road length. Results indicated that on road surfaces employing dispersed drainage, WFD is approximately 6% higher at sag vertical curves and lower at crest vertical curves compared to constant slope segments. Moreover, WFD increases by over 35% at superelevation transitions. To quantify the impact of rainfall on road safety, a critical WFD parameter was developed. This parameter defines the maximum WFD under specific rainfall conditions that reduces the pavement-tire tangential friction coefficient to a level corresponding to the standard stopping sight distance. Using the DE-WFD model, simulations of hourly rainfall intensity and duration identified conditions under which WFD reaches this critical value for various roadway geometries. These findings provide valuable references for the precision management of highway operational safety. This suggests that traffic safety authorities should implement warning and intervention measures when critical rainfall conditions are exceeded to ensure driving safety.

## 1 Introduction

The integration of advanced technologies such as the Internet of Things, artificial intelligence, and computer communications with traditional expressway systems is driving the development of smart expressways that ensure all-weather safe access. A critical challenge is maintaining driving safety during adverse weather, particularly rain. If rainwater is not promptly drained from the road surface, the resulting water film can generate lift on vehicle tires, reducing tire-road contact. This may cause the entire tire track to be supported by a thin water film [1], significantly increasing the risk of vehicular loss of control and posing a severe safety hazard. Statistical evidence underscores the gravity of this issue, showing that collision rates in rainy conditions are over 20% higher than in dry conditions, with injury rates exceeding 40% [2, 3]. Recent findings by the FHWA further emphasize the impact, with rainfall-related collisions causing more than 357,300 injuries and over 3,400 fatalities annually [4]. Analyses of the causes of these collisions have identified the water film as a significant contributing factor [3, 5, 6]. Consequently, accurately predicting WFD is fundamental for ensuring all-weather safe access on smart expressways and for enhancing the precision management of expressway operational safety.

The concept of WFD refers to an undisturbed layer of water above the peaks of pavement roughness. To mitigate the potential threat of water film on road safety, extensive research on WFD prediction has been undertaken. Conventional WFD prediction techniques are generally divided into empirical and analytical methods. The empirical method employs data collected from artificial or natural rainfall experiments to fit predictive models [7–10]. These models establish relationships between slope, runoff length, texture depth (TXD), rainfall intensity, and WFD. However, their applicability is limited by the range of parameters and values within the existing dataset. If road geometric features and rainfall conditions extend beyond the current database, the models’ predictions may become inaccurate. Moreover, the accuracy of empirical models heavily relies on measurement techniques, with common technologies such as point gauges, contact sensors, and optical image processing providing resolutions ranging from 0.1 to 1 mm [8, 11, 12]. In contrast, the analytical method is not limited by data availability and assumes a steady one-dimensional flow on a uniform slope. It constructs mathematical models using equations like the Manning-Chezy formula or the shallow water equation [13, 14]. However, actual road alignments do not always maintain a unidirectional constant slope. For instance, the slope of superelevation transitions and vertical curves can vary with the road’s width or length. Jia et al. [15] analyzed the runoff characteristics of superelevation transitions and found that variations in road cross-slope along the route can cause the path of surface runoff to flow towards the inner lane and then reverse back to the outer lane after crossing sections with zero cross-slope, significantly differing from the assumed unidirectional slope runoff characteristics. Recently, Han et al. [9] focused on WFD in segments with non-constant grades, deriving the WFD for sag vertical curves based on road geometric features. However, this study overlooked the dynamic characteristics of water flow. Consequently, current research on WFD prediction faces several limitations: i) Despite advancements in measuring WFD to a resolution of 0.1mm, this resolution remains insufficient for water films only a few millimeters thick, particularly under weak rainfall intensities. ii) Traditional models often simplify road surfaces to unidirectional uniform slopes, potentially leading to discrepancies between predicted outcomes and actual conditions. iii) Most models fail to reflect the dynamic evolution of WFD during rainfall.

Internationally, the friction coefficient of wet pavements is universally accepted as the fundamental parameter for establishing highway alignment design values [16, 17]. During the 1980s and 1990s, German researchers systematically evaluated tire-pavement friction coefficients under varying surface conditions (dry, wet, and snow-covered) using the Stuttgarter tribometer and PIARC European standard tires. Considering the diversity of pavement types and the effects of cumulative traffic-induced polishing, the 95th percentile friction coefficient, derived from the cumulative frequency distribution curve, was adopted as the standard value [18]. This standard ensures road safety in most scenarios by providing the foundation for establishing critical parameters such as stopping sight distance and curve radius. This implies that as long as the WFD on the road surface during rainy conditions does not exceed the threshold for wet conditions, the highway is likely to provide adequate safety margins for vehicles, thereby posing no substantial threat to road safety. Nevertheless, there remains a lack of consensus among road safety scholars regarding the precise definition of wet conditions. Besse [19] analyzed the relationship between the effective pavement friction coefficient and WFD through field tests finding that the coefficient stabilized at speeds between 32–80 km/h when WFD exceeded 0.508 mm, suggesting this depth as the critical value for the wet state. In contrast, Do et al. [20] conducted experimental studies on thin water films with WFD less than 1 mm, identifying a rapid decline in the effective pavement friction coefficient starting at a critical WFD of 0.21 mm. Kulakowski et al. [21] systematically collected data using a specially designed device, revealing an approximately exponential decrease in the effective friction coefficient with increasing WFD. They introduced a parameter of pavement moisture sensitivity, selecting 75% of this sensitivity as the standard for establishing critical WFD, although this standard is considered overly subjective and lacks theoretical support. In summary, further analysis of the critical WFD that characterizes pavement wet conditions is warranted, specifically in: i) developing an evaluation method for critical WFD related to road design values, which can intuitively reflect the impact of rainfall on road safety, and ii) determining the rainfall conditions under which WFD reaches critical values for various highway alignments, thus facilitating timely interventions by traffic management authorities.

Recent advancements in BIM and Computational Fluid Dynamics (CFD) technologies have opened new pathways for comprehensive analysis of WFD. BIM technology offers more precise replication of road geometric features compared to conventional LiDAR scanning techniques [10], providing a robust foundation for predicting WFD across various highway alignments. Conversely, CFD technology has shown substantial potential for evaluating film thickness and excels in modeling the formation and evolution of liquid films, outperforming conventional methods [22, 23]. Integrating these technological benefits, this study employs BIM to accurately reproduce the 3D roads under typical alignment combinations and combines it with CFD to simulate the formation and development of water films, thereby aiming to deliver more precise predictions of WFD.

In a nutshell, the contributions of our work are:

- A DE-WFD model utilizing BIM and CFD technologies is developed. This model accurately predicts WFD across various highway alignments and effectively captures variations in WFD induced by changes in longitudinal and cross slopes.
- Furthermore, a method for estimating critical WFD is proposed. This method determines the maximum WFD under specific rainfall conditions that reduces the tire-road tangential friction coefficient to the value corresponding to the standard stopping sight distance. It effectively reflects the impact of rainfall on road safety and provides a reference for traffic management authorities to implement timely intervention measures.
- Lastly, the necessary rainfall intensity and duration for WFD to reach critical values under various road geometric conditions are identified.

This paper is organized as follows: Section 2 describes the meticulous reproduction of 3D roads using BIM and mesh division technologies, along with the development of the DE-WFD model based on the discrete phase model-Eulerian wall film coupling. Section 3 evaluates the validity and accuracy of the DE-WFD model in terms of grid independence and WFD. Section 4 presents and discusses the results, and finally, Section 5 concludes the paper and outlines future work.

## 2 Materials and methods

During rainfall events, the formation of a water film involves multiple physical interactions between rainwater and the pavement. Initially, raindrops striking the road surface cause a splashing phenomenon dispersing rain particles and generating minute droplets. At this stage, the rainfall has not yet exceeded the pavement’s infiltration and texture storage capacity, so no water film forms. However, once the volume of water surpasses these capacities, excess water begins to pool and flow, ultimately resulting in the formation of a continuous water film. The complex dynamics of water film formation and development present significant challenges for in-field measurements. For example, the diversity and complexity of highway alignments complicate the accurate assessment of water film distribution and thickness prediction, particularly in segments where slopes vary along the road length. Nevertheless, with advancements in BIM and CFD technologies, it is now feasible to precisely delineate road geometric features and accurately simulate film thickness. Although seldom applied to predict WFD, these technologies have been effectively utilized and validated in studies such as slag film formation mechanisms [24] and the reproduction of cough droplet formation processes [25]. This paper employs these advanced technologies to reconstruct the complex physical processes involved in water film formation and development.

### 2.1 Designing 3D road models

The design of 3D road models involves two key phases: determining highway alignments and modeling the roads.

Determining of highway alignments is fundamental to road modeling. This study employed a systematic approach to define highway alignments, enabling a comprehensive assessment of their impact on WFD. Highway alignments were categorized into four types based on variations in grade and cross-slope along the road length: (i) segments with constant longitudinal and cross slopes, including straight slopes, circular curves, and their combinations; (ii) vertical curves with varying longitudinal slopes and constant cross slopes; (iii) superelevation transitions with constant longitudinal slopes and varying cross slopes; and (iv) sections with variable longitudinal and cross slopes in superelevation transitions and vertical curves. The fourth category is often avoided in highway alignment design due to drainage issues [16]. Therefore, this research focused primarily on the first three categories. Table 1 presents the road alignment parameters used for road modeling.

**Table 1:** Summary of road alignment parameters used for road modeling.

Type	i	ii	iii
**Grade (*i***_***g***_**, m/m)**	±0.005, ±0.015, ±0.03, ±0.045	-	The same as Type i
**Vertical Curve Radius (*R*, m)**	-	3000, 6000, 9000	-
**Vertical Curve Type (VCT)**	-	Sag, Crest	-
**Superelevation (*i***_***h***_**, m/m)**	±0.02, ±0.04, ±0.06	The same as Type i	-
**Superelevation Transition Rate (*p*)**	-	-	1/200, 1/250, 1/330
**Roadway Width (*W*, m)**	18		
**Road Length (*L*, m)**	Eq (1)		

Note: A positive ’*i*_*g*_’ indicates an uphill slope, whereas a negative ’*i*_*g*_’ denotes a downhill slope. A positive ’*i*_*h*_’ signifies that the elevation of the outer edge of the median separator is higher than that of the edge of the outer traffic lane. Conversely, a negative ’*i*_*h*_’ indicates a lower elevation of the median separator’s outer edge relative to the edge of the outer traffic lane.


$$L=\left\{\begin{array}{l} \frac{\alpha_{\text{i}}wi_{g}}{i_{h}},\;\text{for}\;\text{Type}\;\text{i}\\ \left(i_{gb}-i_{gf}\right)R+2L_{\text{ii}},\;\text{for}\;\text{Type}\;\text{ii}\\ \frac{\left(w_{l}n_{w}\right)\;\Delta_{i}}{p}\xi_{w}+2L_{\text{iii}},\;\text{for}\;\text{Type}\;\text{iii} \end{array}\right.$$ (1)

where, *α*_i_ represents the extension coefficient for Type i. *L*_ii_ and *L*_iii_ denote the extended lengths of the preceding and following slopes for Type ii, and the lengths of the normal cross slope segment and full superelevation segment for Type iii, respectively. *i*_*gb*_ and *i*_*gf*_ are the gradients of the slopes preceding and following the vertical curve, respectively. *w*_*l*_ is the width of the lane. *n*_*w*_ represents the number of turning lanes. *ξ*_*w*_ is the adjustment factor for the number of turning lanes, calculated as ξ_*w*_ = [1+0.5(*n*_*w*_-1)]/*n*_*w*_ [16]. Finally, *Δi* is the algebraic difference between the designed superelevation and the normal cross slope.

The highway alignment parameters in Table 1 are derived from the ranges recommended by the Technical Standard of Highway Engineering [17]. The road length includes both a basic length and an extended length. The basic length, based on existing literature [9], denotes the minimum distance necessary to ensure a complete distribution of the water film. The extended segment is designed to accommodate potential uncertainties and variability, ensuring comprehensive data collection on water film distribution. The road models considered only the width of the right roadway (left curb offset 0.5 m + traffic lanes 4×3.75 m + traffic shoulder 2.5 m), excluding the earth shoulder and roadside barriers. These exclusions were justified because rainfall on two-way lanes does not converge but is instead directed by the drainage systems of the central median separator and the lateral ditches or slopes. Consequently, only the width of one half of the roadway was considered. Furthermore, the cross-slope of earth shoulders is generally greater than that of the lanes and hard shoulders, directing the flow of rainwater predominantly towards the subgrade slopes or roadside ditches. Consequently, the impact of earth shoulders on water accumulation in lanes can be virtually disregarded. Similarly, roadside barriers have an insignificant effect on the WFD and were omitted from the model.

**Fig 1:**
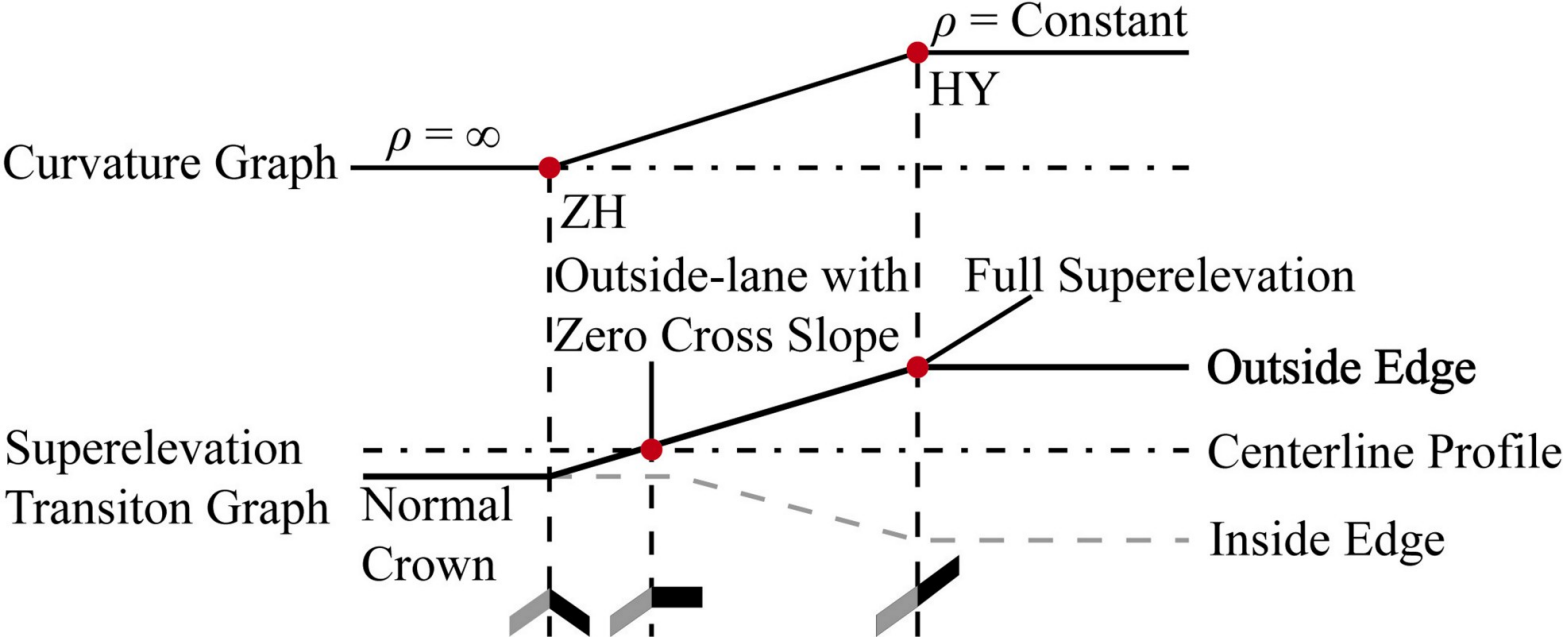
Schematic diagram of Type iii superelevation transition.

**Fig 2:**
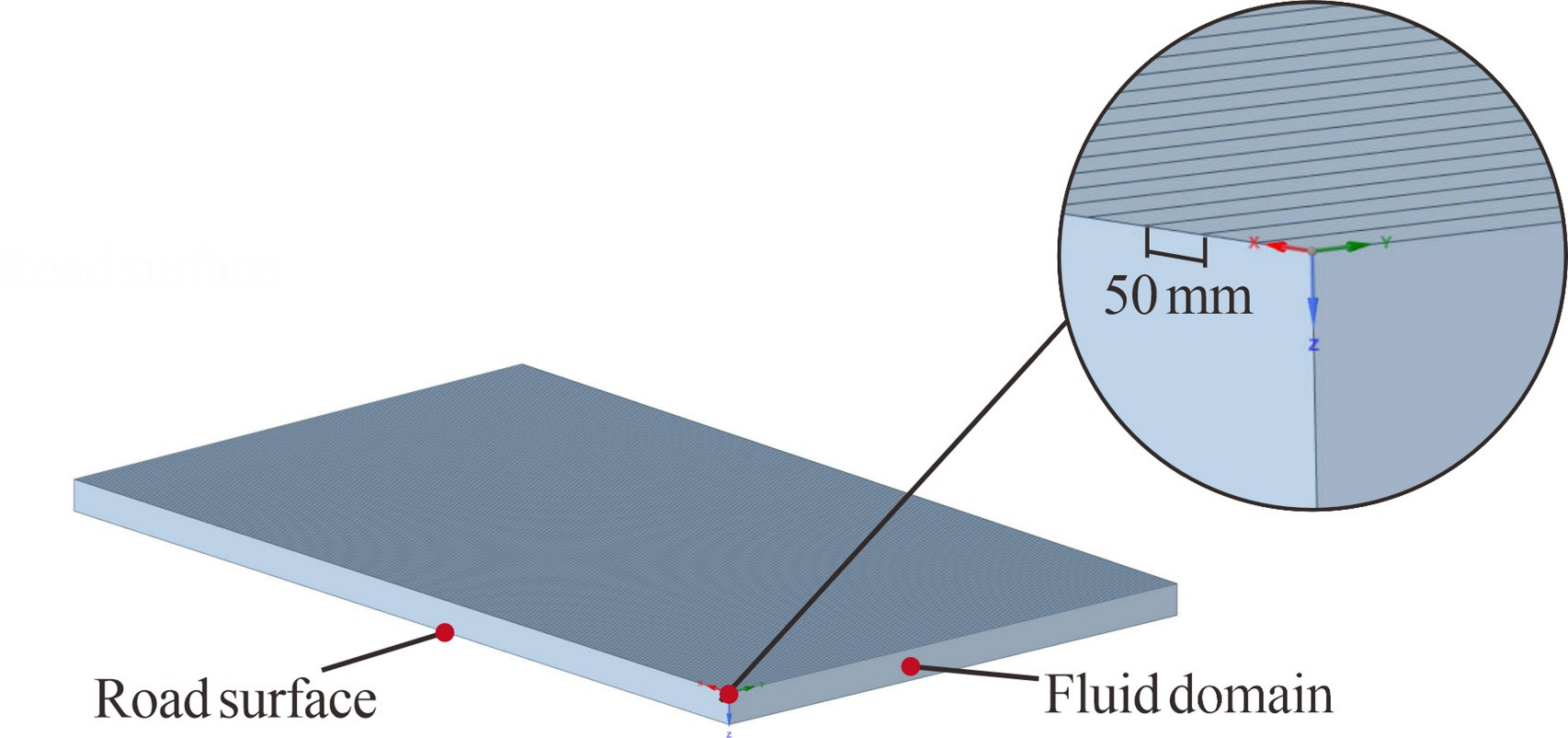
3D model of the road.

The construction of 3D road models in the BIM modeling software, OpenRoads Designer^®^, was completed in two principal steps. First, the design was developed sequentially through horizontal alignment, vertical alignment, and cross-section, guided by the parameters listed in Table 1. For Type iii, a superelevation transition design, employing methods used in countries such as China and Japan, was required. This transition starts at the tangent-to-spiral (ZH) junction, rotates the traveled way about the centerline profile, and ends at the spiral-to-curve (HY) transition at the circular curve’s end, as depicted in Fig 1. The second step involved generating the 3D road models based on the design outcomes. OpenRoads Designer^®^ constructs these models using polylines, maintaining a 50 mm interval between points to ensure the slope error remains below 0.0016%. Additionally, to facilitate rainfall simulation, a fluid domain measuring 1 m in height was created above the road surface, as detailed in Fig 2.

In this study, dispersed drainage was employed as the roadway drainage approach, commonly used in highway drainage design for subgrade slopes that are not susceptible to erosion. Notably, concentrated drainage serves as an alternative, typically implemented where subgrade slopes are prone to erosion. The design of concentrated drainage considers the configuration, placement, and spacing of drainage facilities. These factors significantly influence the efficiency of roadway runoff, thereby impacting the WFD [26, 27].

### 2.2 Meshing

The mesh delineates the geometric characteristics of the computational domain and directly influences the accuracy of WFD simulations. Consequently, the type, size, and quality of mesh elements determine the precision and reliability of WFD simulation results.

Hexahedral meshes were utilized due to their advantages over tetrahedral finite element meshes, including reduced errors, fewer elements, and enhanced reliability. These qualities contribute to more stable numerical performance and greater computational efficiency [28].

The mesh size critically affects both the fidelity of 3D road simulations and the precision and computational costs incurred. Utilizing smaller mesh sizes allows for a more detailed depiction of highway geometric features and finer detail resolution in flow fields; however, it significantly increases computation time and resource requirements. To optimize the balance between accuracy and efficiency, the global mesh size for the road 3D model was set at 50 mm. Given the considerable impact of wall effects on fluid dynamics and significant velocity gradients, densifying the boundary layer is imperative to maintain modeling quality. In this paper, the initial mesh height was established at 0.1 mm with a growth rate of 1.1, ensuring a smooth transition across mesh heights. The adequacy of these mesh sizes was confirmed through mesh independence tests.

**Fig 3:**
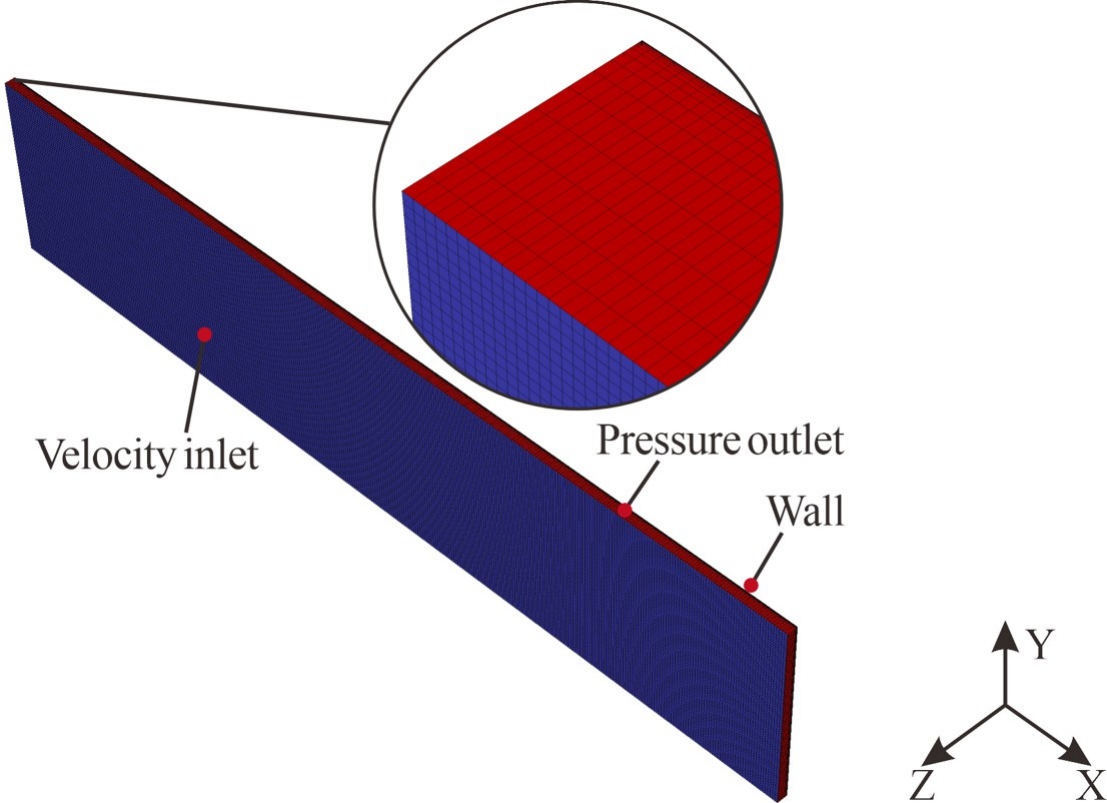
An example of road model meshing.

The geometry of mesh elements significantly influences simulation outcomes. Ideally, these elements should be regular and maintain orthogonality to minimize potential errors. Distorted or elongated mesh elements often result in increased numerical errors and reduced convergence rates. The determinant, a critical metric for assessing mesh quality, calculates the ratio of the smallest to the largest determinant of the Jacobian matrix at each element’s node [29]. A determinant value of 1 signifies a perfectly regular mesh element, whereas a value of 0 indicates a degenerate element in one or more edges, and negative values reflect inverted elements. In the models evaluated, all determinant values exceed 0.9, demonstrating that the mesh elements are of exceptional quality. Fig 3 illustrates the mesh partitioning results for one of the simulated conditions.

### 2.3 DE-WFD modeling

Previous research on WFD often overlooked the formation and evolution of the water film, potentially leading to biased WFD estimates. To address this gap, we introduced a discrete phase model coupled with the Eulerian wall film model to predict WFD (referred to as the DE-WFD model). In this model, raindrop precipitation was simulated using the discrete phase model, while the flow properties of the water film were characterized by the Eulerian wall film model. Notably, the integrated model incorporated the following physical processes: (i) the inlet fluid is a mixture of gas and water droplets, (ii) the splashing and stripping of water droplets, and (iii) bidirectional coupling between the water film and the gas phase.

The discrete phase model (DPM) is a numerical method used to simulate the dynamics of discrete entities, such as particles, droplets, and bubbles, within a continuous fluid medium. Its theoretical foundation is based on the equation of motion derived from Newton’s second law, describing the trajectory of particles influenced by external forces, including gravity and aerodynamic drag, as shown in Eq (2) [30]. Since raindrops fall as discrete particles rather than forming a continuous liquid phase, the DPM effectively models their behavior within a continuous medium. Consequently, the DPM is used to simulate the rainfall process.

$$\frac{d{\vec{u}}_{p}}{dt}=\frac{18\mu \left(\vec{u}-{\vec{u}}_{p}\right)}{\rho_{p}d_{p}^{2}}\frac{C_{D}Re_{p}}{24}+\vec{g}\left(1-\frac{\rho }{\rho_{p}}\right)$$ (2)

where *${\vec{u}}_{p}$* represents the velocity of the particle, *$\vec{u}$*denotes the flow velocity of the continuous phase, *μ* signifies the viscosity of the continuous phase, *ρ*_*p*_ is the density of the particle, *d*_*p*_ refers to the diameter of the particle, *C*_*D*_ is the drag coefficient, *Re*_*p*_ is the Reynolds number for the particle, $\vec{g}$indicates gravitational acceleration, and *ρ* is the density of the continuous phase.

Raindrops impacting the roadway generate a flowing water film influenced by wall shear stress, air pressure, surface tension, and the film’s viscosity. Under specific conditions, surface tension may cause the separation of the water film, resulting in rivulet formation. The behavior of the water film is simulated using the Eulerian wall film (EWF) model. This model captures the complex physical processes involved in the formation, flow, diffusion, and evaporation of the water film and has been successfully utilized in various fields including energy [22, 31] and medicine [25, 32]. Typically, the WFD is relatively thin, justifying the neglect of flow perpendicular to the road surface. Furthermore, the flow of the water film is primarily driven by gravity, wall friction, and surface tension, with wind effects considered negligible. Based on these assumptions, the equations for mass, momentum, and energy conservation of the water film are established, as shown in Eq (3) [30]. These equations account for various physical effects, including surface tension, viscous resistance, gravity, and interactions with particles, detailing changes in WFD, velocity, and temperature.

$$\left\{\begin{array}{l} \frac{\partial \rho_{f}\delta }{\partial t}+\nabla \cdot \left(\rho_{f}\delta v_{f}\right)=\delta \left(M_{pf}-M_{f}\right)\\ \frac{\partial \rho_{f}\delta v_{f}}{\partial t}+\nabla \cdot \left(\rho_{f}\delta v_{f}v_{f}\right)=-\delta \cdot \nabla P_{L}+\delta \rho_{f}{\vec{g}}_{\tau }+\frac{3}{2}\tau_{g-f}-\frac{3\mu_{f}}{\delta }v_{f}+\delta \left(M_{pf}-M_{f}\right)v_{f}\\ \frac{\partial \rho_{f}\delta h_{f}}{\partial t}+\nabla \cdot \left(\rho_{f}\delta h_{f}v_{f}\right)=\frac{\lambda_{\text{f}}}{\delta }\left(T_{s}+T_{w}-2T_{m}\right)+\delta \left(M_{pf}-M_{f}\right)h_{v} \end{array}\right.$$ (3)

with,

$$p_{L}=p_{g}-\rho \delta (\vec{n}\cdot \vec{g})-\nabla \sigma \cdot (\nabla \delta )$$ (4)

where *ρ*_*f*_ represents the density of the liquid film, while *δ* denotes the thickness of the film. *M*_*pf*_ is the mass source term between the water droplets and the film. *${\vec{g}}_{\tau }$*corresponds to the component of gravity parallel to the water film. *τ*_*g*-*f*_ refers to the viscous shear stress at the gas-film interface. *μ*_*f*_ is the dynamic viscosity of the film. *h*_*f*_ indicates the enthalpy of the film. *T*_*s*_, *T*_*w*_ and *T*_*m*_ represent the temperatures at the surface of the water film, the road surface, and the mid-depth of the water film, respectively.

### 2.4 Solution strategy

The upper surface of the 3D road models was designated as the inlet, with the periphery defined as the outlet. Velocity inlet and pressure outlet boundary conditions were applied at these boundaries, respectively. Droplets expelled through the pressure outlet were considered to have escaped. The inlet temperature was set at 300 K, with relative humidity maintained at 100%. Droplets were released at the inlet with an initial velocity consistent with the gas phase, calculated using the terminal velocity formula for droplets as indicated in Eq (5) [33]. This formula had been validated by spectrometer experiments and effectively captured the velocity of droplets impacting the road surface [34]. The median value indicated in Eq (6) was used as the typical droplet diameter, following observations of a statistically significant correlation between median droplet diameter and rainfall intensity during precipitation events [35]. Further studies indicated that droplet diameter distribution characteristics minimally impact the flow behavior of rainfall [36]. Therefore, the distribution characteristics of raindrop diameters were simplified to a uniform distribution. The mass flow rate of the droplets was calculated based on rainfall intensity and road surface area, as depicted in Eq (7).

$$u\left(D\right)=0.0000561D^{3}-0.00912D^{2}+0.503D-0.254$$ (5)

$$D_{50}=0.97I_{w}^{0.158}$$ (6)

$$\text{TF}=\frac{I_{w}S_{r}}{3600}$$ (7)

where *u* represents the terminal velocity of raindrops, *D* denotes the diameter of the raindrops, *I*_*w*_ indicates the intensity of rainfall, *S*_*r*_ refers to the surface area of the simulated roadway, and TF is the mass flow rate.

The wall served as a boundary condition for the road surface, where impacting raindrops are collected into a water film, fragmented, or rebounded. Two textural depths (TXDs) of the pavement, specifically 1.2 mm and 0.55 mm, were considered to evaluate the influence of pavement polishing on roadway runoff [37, 38]. This highlights that TXD decreases over time due to repeated vehicle loading [37, 39]. Importantly, the rate of TXD deterioration is influenced not only by the cumulative number of axle load applications but also by the properties of the pavement material. For example, degradation rates vary between asphalt and concrete pavements and among similar pavements composed of different materials.

All simulations were conducted using Ansys Fluent^®^. The steady pressure-based solver was employed to calculate the governing equations. The RSM turbulence model was utilized for its high precision in turbulent problems [40]. The Coupled algorithm was used to couple the velocity and pressure fields. A second-order upwind scheme was adopted for discretizing the governing equations. Convergence was considered achieved when the residual of the WFD fell below 1×10^−5^, indicating the stabilization of the WFD.

## 3 Results and discussion

The objective of this study is to develop a WFD prediction method that is both broadly applicable and highly accurate, while also identifying the rainfall intensity and duration that significantly impact vehicular safety. To accomplish this, field experiments and classical WFD prediction models are first introduced to compare and validate the accuracy of the DE-WFD model. Subsequently, the advantages of the DE-WFD model over traditional models are highlighted by demonstrating its sensitivity to variations in WFD resulting from changes in road slope and superelevation. Moreover, the spatiotemporal distribution and variations in WFD across different three-dimensional pavement configurations are analyzed. Finally, by introducing the critical WFD index, the rainfall intensity and duration necessary to reach the critical WFD for pavements with various alignment combinations are determined, providing crucial reference data for the refined and proactive management of smart highway safety during rainy conditions. The detailed data related to the figures and tables presented in this paper are described in S1 File.

### 3.1 Verification of WFD accuracy

**Fig 4:**
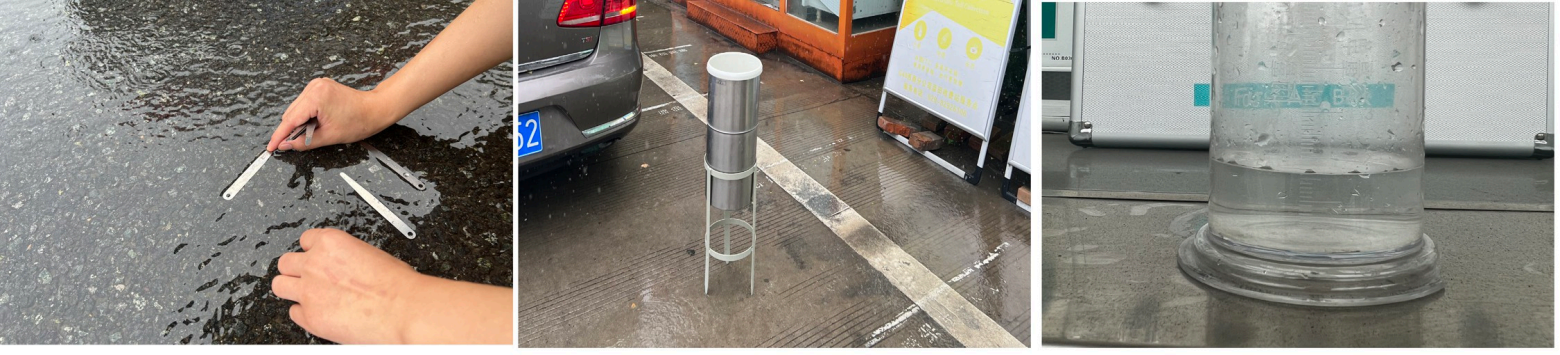
The test personnel were measuring the rainfall intensity and WFD. (a) WFD was being measured on-site using a feeler gauge. (b) Rainfall was being collected on-site using a rain gauge. (c) Rainfall intensity was being measured on-site using a measuring cylinder.

The accuracy of the DE-WFD model was validated by comparing it with field tests and conventional models. The procedure for the field tests is outlined below:

- Selection of test sites: Six test sites were chosen along the G30, G40, and G70 expressways in China. Due to the spatial and temporal uncertainties of rainfall and the potential risks associated with conducting tests on busy roadways, the locations were selected flexibly based on real-time rainfall conditions and traffic volume. Observations were conducted under free-flowing traffic conditions to ensure the safety of experimental personnel and minimize the impact of traffic flow on WFD measurements. Additionally, retrospective analysis of cloud monitoring videos indicated that rainfall at the observation points exceeded 1.5 hours, suggesting that the pavements were in a state of wet saturation.
- Measurement of WFD (Fig 4(A)): The measurement points were positioned 0.2 meters from the edge of the right lane marking, and the WFD was measured using the stacked feeler gauge method [41]. The measurement concluded when the second-to-last gauge was submerged without submerging the top 0.01 mm gauge, ensuring precision within 0.01 mm. To mitigate errors from rain splash, an umbrella shielded the test area throughout the procedure. To further reduce systematic errors, the process was repeated five times, and the average value was recorded as the final WFD. Additionally, measurements were taken 0.2 meters inside the lane marking to avoid disturbances from the lane markings, which are often painted with thermoplastic material varying in thickness between 0.7 mm and 2.5 mm. Upon completion, the corresponding pile number for each test site was recorded.
- Measurement of rainfall intensity (Fig 4(B) and 4(C)): While measuring WFD, continuously measure the 15-minute rainfall using a rain gauge at safe locations near the test road section, such as toll plazas and service areas. Convert the readings from the measuring cylinder into hourly rainfall intensity.
- Examination of highway alignment, pavement structure, and TXD at measurement points: We determined the highway alignment and pavement structure at the measurement points using pile numbers from the original design documents. The pavement structure at these points is either AC-13 or SMA-13, with a void ratio of 3–4.5%. At this void ratio, the pavement permeability coefficient is very small and can be ignored [42, 43]. Additionally, we established the TXD at these points by reviewing the most recent road maintenance reports.

Based on the described experimental protocol, the WFD measurements from six observation points are detailed in Table 2. The data reveal a notably low standard deviation, statistically confirming the consistency and stability of the findings. Furthermore, even at minimal rainfall intensities of 1.5 mm/h, the ratio of the measurement resolution (0.01 mm) to the actual values remains below 5.5%, emphasizing the accuracy of the employed method. Therefore, this paper substantiates the feasibility and increased precision of using the feeler gauge for WFD measurements.

**Table 2:** Measurement values of WFD at each test site.

Test site	TXD (mm)	Grade	Superelevation	Distance to the outer edge of the median separator (DTC, m)	Rainfall intensity (mm/h)	Measured WFD (M-WFD, mm)
**1**	0.81	0.013	-0.02	11.55	1.5	0.184±0.0037
**2**	1.02	0.009	-0.02	3.6	6.2	0.316±0.0023
**3**	0.91	0.015	-0.02	8	4.65	0.332±0.0021
**4**	1.10	0.043	0.04	7.8	10.2	0.564±0.0239
**5**	0.83	0.014	-0.02	11.55	7.8	0.690±0.0032
**6**	0.83	0.014	-0.02	15.3	7.8	0.852±0.0057

Note: The test sites are for dispersed drainage with efficient drainage.

Several representative conventional models were selected for comparative analysis with the DE-WFD and empirical values: the RRL model [7], the Gallaway model [8], and the PAVDRN model [44]. Developed by the British Road Research Laboratory, the RRL model is based on indoor rainfall simulation test data. The Gallaway model, devised for the U.S. Department of Transportation by Gallaway et al., describes the relationship between WFD, TXD, longitudinal and cross slopes, and rainfall intensity using empirical data. The PAVDRN model, a computer program developed with NCHRP funding by the University of Pennsylvania, employs a one-dimensional steady-state form of the kinematic wave equation to calculate WFD. Details are provided in Table 3.

**Table 3:** Summary of conventional WFD prediction models.

Model	Equation
**RRL (R-WFD)** [7]	$\text{WFD}=0.015{\left(L_{\text{f}}+I_{\text{r}}\right)}^{0.5}i_{\text{f}}^{-0.2}$, $i_{\text{f}}=\sqrt{i_{\text{g}}^{2}+i_{\text{h}}^{2}}$, $L_{\text{f}}=W\times \frac{i_{\text{f}}}{i_{\text{h}}}$
**Gallaway (G-WFD)** [8]	$\text{WFD}=0.01485\left(\frac{{\text{MTD}}^{0.11}\times L_{\text{f}}^{0.43}\times I_{\text{r}}^{0.59}}{i_{\text{h}}^{0.42}}\right)-\text{MTD}$
**PAVDRN (P-WFD)** [44]	$\text{WFD}={\left[\frac{nL_{\text{f}}I_{\text{r}}}{105.425i_{\text{f}}^{0.5}}\right]}^{0.6}-\text{MTD}$

In Table 3, the symbols represent the following variables: *I*_r_ denotes rainfall intensity, *W* represents the width of flow convergence, *L*_f_ indicates runoff length, *i*_h_ is the cross slope, *i*_g_ refers to the grade, *i*_f_ is the resultant gradient, *η* signifies the Manning roughness coefficient (approximately 0.016 for asphalt pavements), and MTD stands for mean TXD.

**Fig 5:**
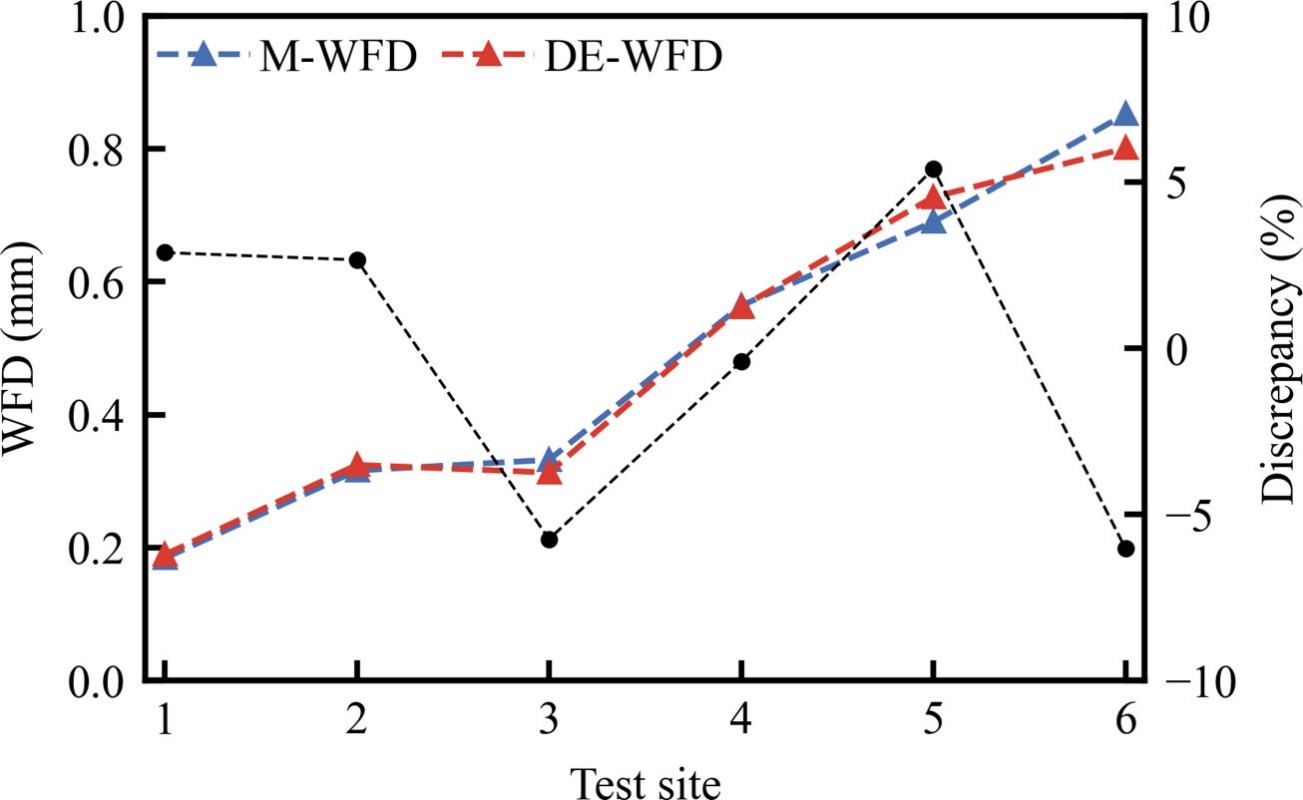
Comparison of DE-WFD with M-WFD.

**Fig 6:**
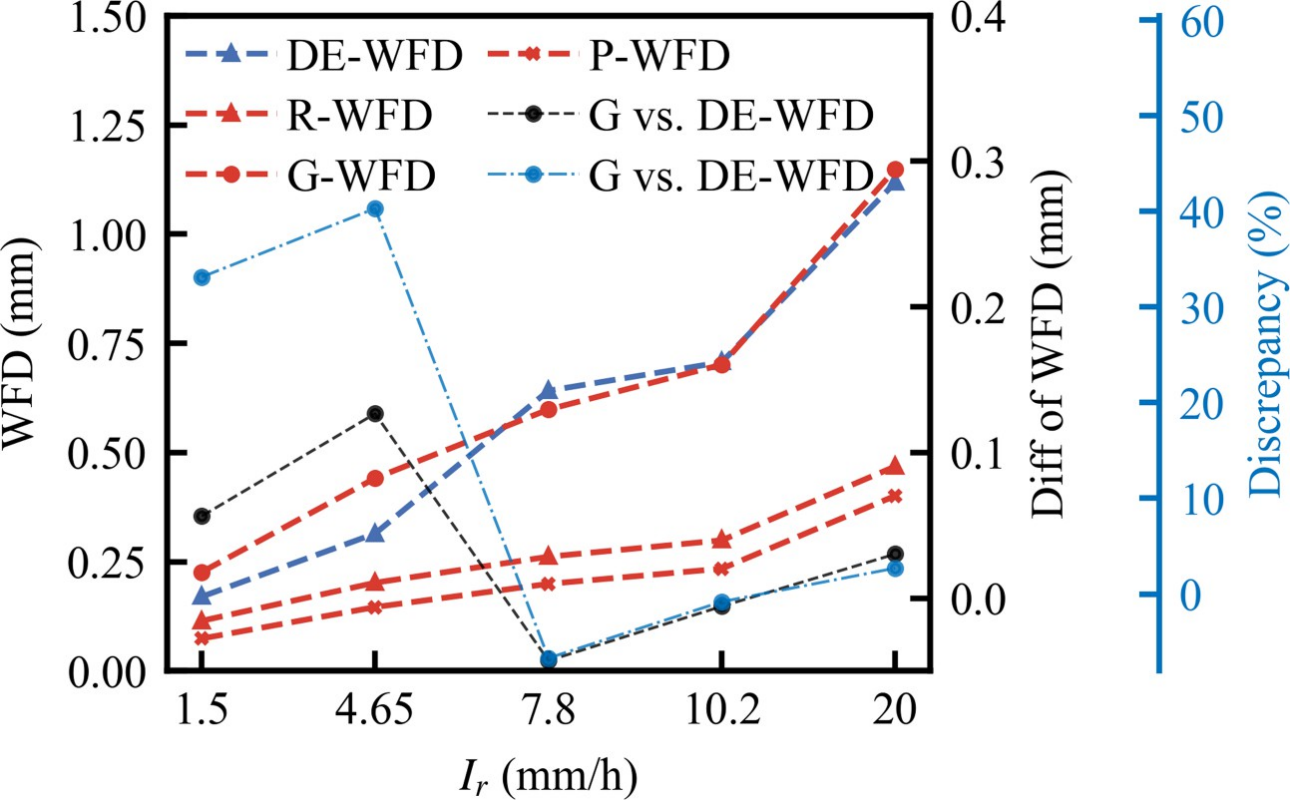
Comparison of DE-WFD with conventional models.

Fig 5 presents the comparative analysis of WFD predictions made by the DE-WFD model against M-WFD. The analysis reveals that the DE-WFD model’s predictions closely align with the actual measurements, exhibiting a maximum deviation of only 7%, highlighting the model’s high precision. Fig 6 further validates the performance of the DE-WFD model by comparing its predictions to those of conventional models under various rainfall intensities. The R-WFD and P-WFD models generally predict lower WFD values than the DE-WFD, likely due to their insufficient consideration of cumulative rainfall effects. Conversely, the G-WFD model predicts higher values than the DE-WFD when the rainfall intensity is below 7.8 mm/h, with a maximum deviation of approximately 0.12 mm (40%). Above this threshold, the predictions of the G-WFD model align closely with those of the DE-WFD. The significant deviation at light rainfall intensities may be attributed to the G-WFD model being calibrated for rainfall intensities up to 51 mm/h, which limits its accuracy at lower intensities. Overall, the DE-WFD accurately reflects the impacts of rainfall intensity and road conditions on WFD, underscoring its advantages in predicting WFD.

### 3.2 Analysis of WFD distribution characteristics

**Fig 7:**
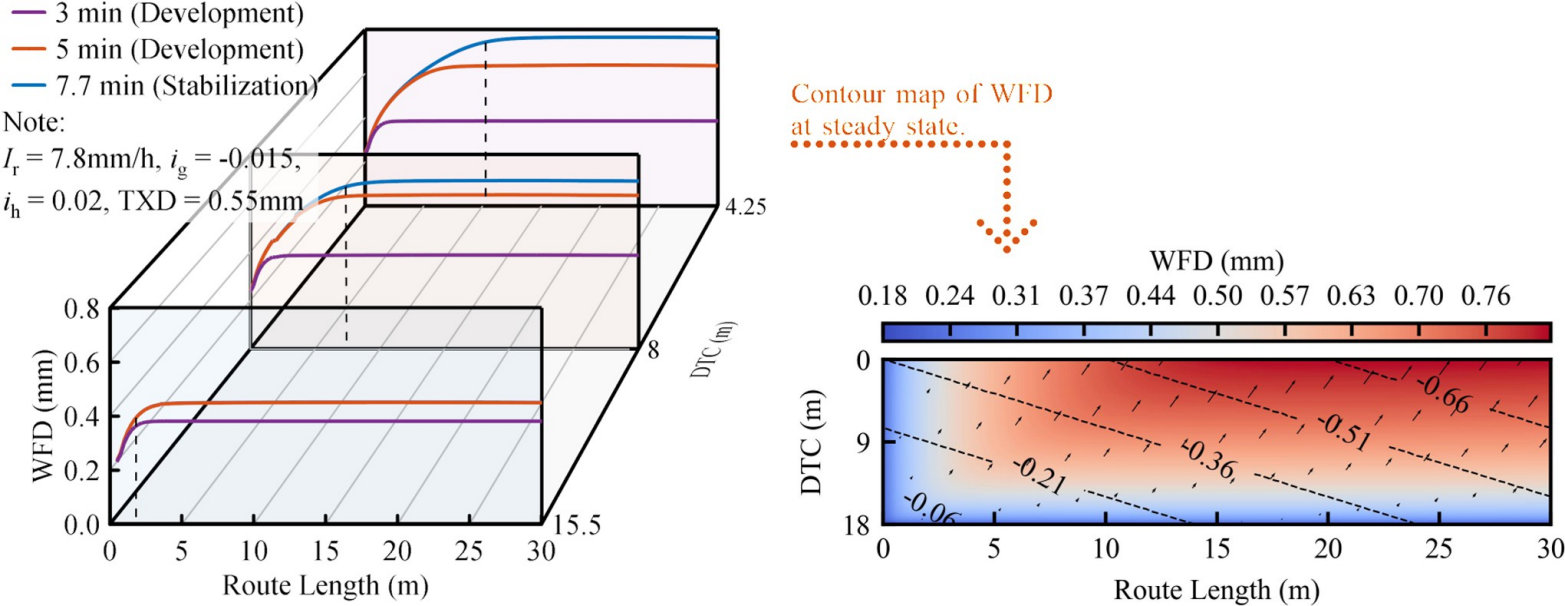
Spatiotemporal distribution characteristics of WFD road segments of Types i.

**Fig 8:**
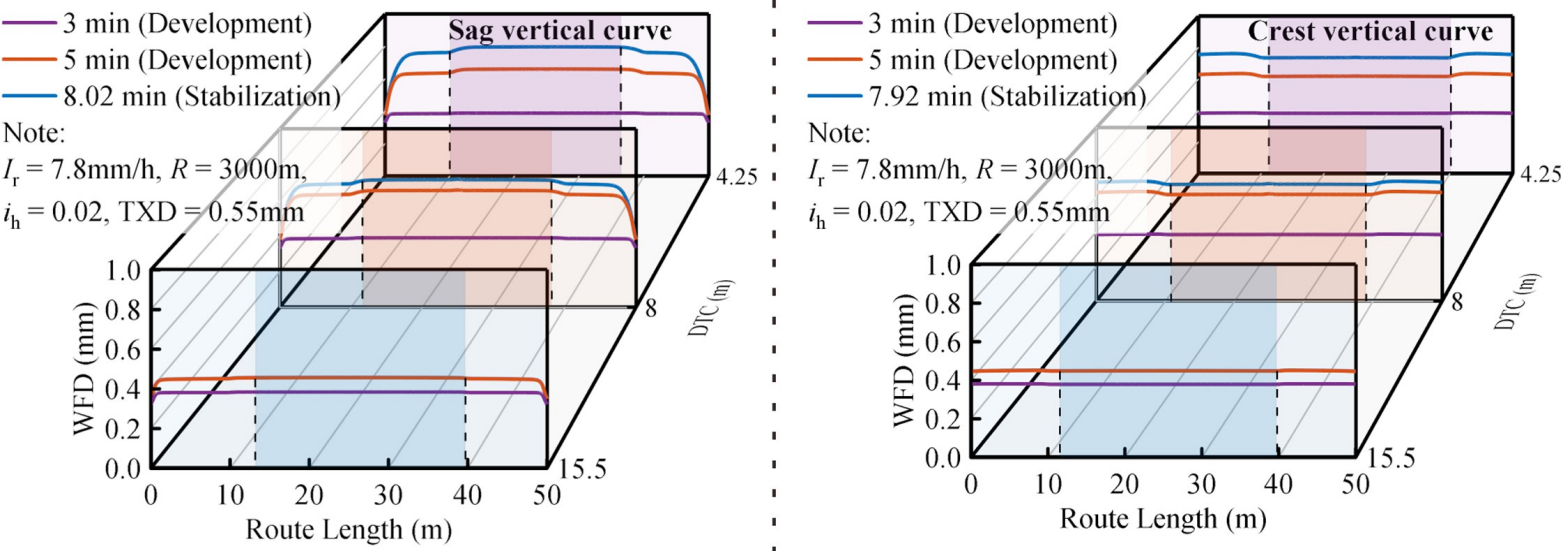
Spatiotemporal distribution characteristics of WFD road segments of Types ii.

**Fig 9:**
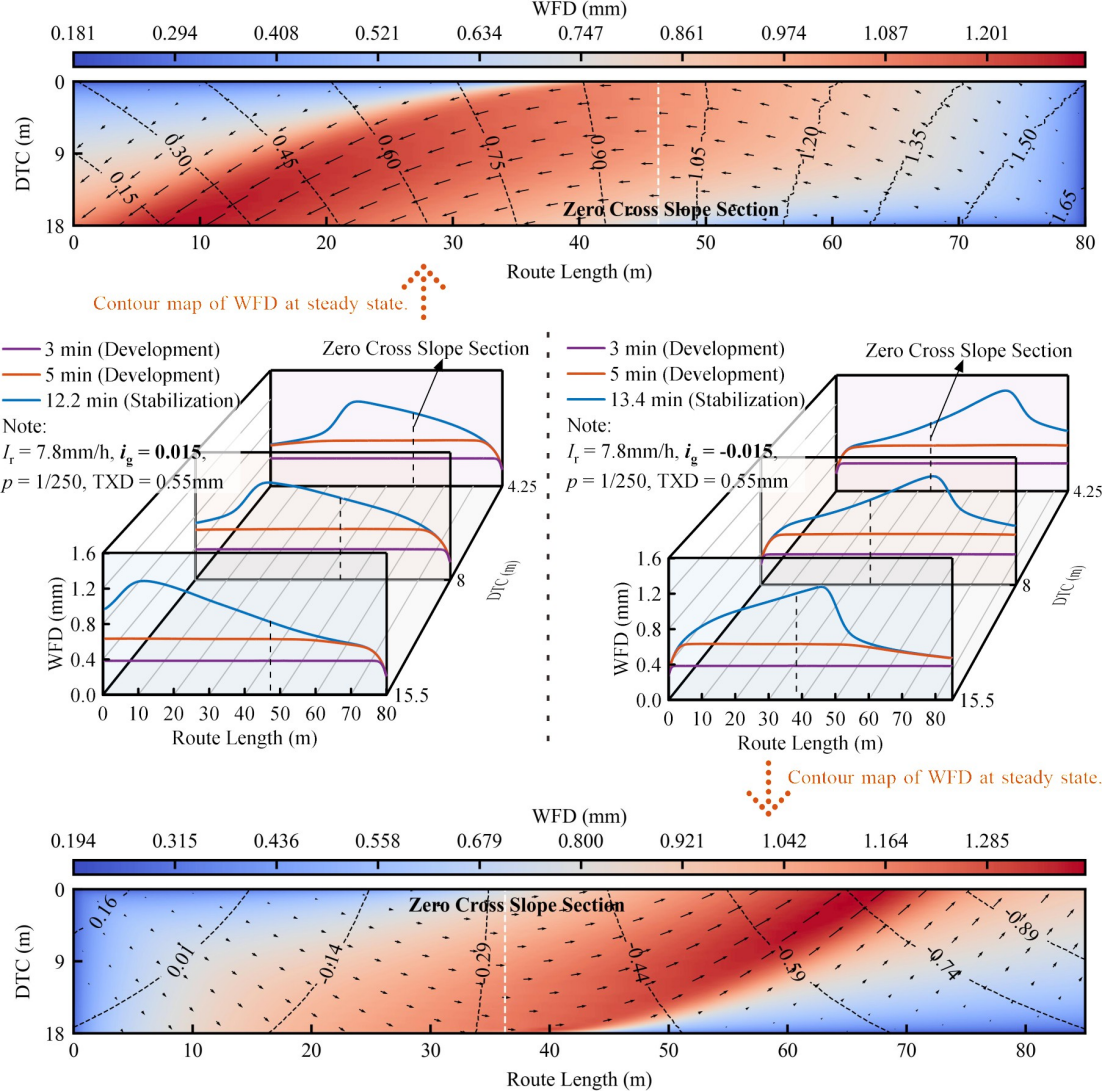
Spatiotemporal distribution characteristics of WFD road segments of Types iii.

To assess the sensitivity of the DE-WFD model to different road alignments, the spatial and temporal distribution characteristics of WFD on road segments of Types i, ii, and iii are illustrated in Figs 7–9. The contour maps in these figures depict the stabilization phase of WFD, where color gradients represent different WFD levels, black dashed lines mark contour lines, and arrows indicate the direction and magnitude of runoff velocity. The results reveal a similar temporal trend across these segments, characterized by an initial increase in WFD followed by stabilization. This trend results from the initial imbalance between rainfall and road surface drainage at the onset of precipitation. As the event progresses, a dynamic equilibrium between rainfall and drainage is established, leading to the gradual stabilization of WFD. However, spatial disparities are evident in the distribution of WFD across different road segments. For Type i, as depicted in Fig 7, the WFD at identical DTC increases incrementally along the route until it stabilizes, a trend consistent with conventional model predictions [7, 8, 44]. Furthermore, this study identified a notable phenomenon: the peak WFD does not appear at the lowest point of the resultant gradient (illustrated by the dashed line in the figure), as typically predicted by conventional models. Instead, the highest WFD is found downstream from this nadir during the stabilization phase. This occurrence is likely due to the transfer of residual kinetic energy within the water flow. As the water passes the gradient’s lowest point, it transfers this energy downslope, where it is gradually dissipated by road surface friction and other resistive forces. Consequently, the WFD that accumulates at this new position exceeds the levels anticipated at the lowest point of the resultant gradient. This phenomenon significantly emphasizes the need to incorporate kinetic energy transfer effects into models to improve the accuracy of road surface WFD predictions.

For road segments of Type ii, the WFD in sag vertical curves follows an increasing, stabilizing, and then decreasing pattern along the road length at consistent DTC, while in crest vertical curves, the pattern reverses to decrease, stabilize, and then increase (Fig 8). Controlling for other factors, the WFD in sag vertical curves is found to exceed that in crest vertical curves. Han et al. [9] conducted one of the few studies investigating the WFD in the road segments of Type ii. Their approach involved modeling the sag vertical curve as an inclined cylinder and conducting a comprehensive analytical study to assess the WFD at the base of these curves. This model is considered appropriate because it effectively captures the essential characteristics of water film accumulation at the lower slope, consistent with our findings. However, the utility of this simplified model is constrained on crest vertical curves due to the opposing runoff direction compared to sag vertical curves, rendering the inclined cylinder model insufficient for accurately depicting the WFD on crest vertical curves. This observation emphasizes the importance of considering specific road alignments in WFD predictions.

Unlike the stable trend observed in the WFD of Type i road sections, Type iii sections demonstrate an initial increase followed by a decrease in WFD along the route under identical DTC, especially when a steady state is reached in the time domain. This phenomenon is attributed to the gradual and directional changes in the cross slope over the length of the route. Additionally, Fig 9 suggests that variations in the grade significantly affect the position of the maximum WFD. Specifically, on downhill slopes, the maximum WFD appears in the outer lane before the section with zero cross-slope, while on uphill slopes, it is located conversely. Extending the findings of [45], which indicated that wider road widths on superelevation transitions correlate with greater WFD, this study reveals that both the position and magnitude of the maximum WFD at these transitions are substantially influenced by the gradient.

In summary, conventional models are inadequate for capturing the temporal dynamics of WFD. Simplifying grade and superelevation as constants along the road length fails to accurately represent the characteristics of water film distribution. Consequently, we further analyzed the deviations in WFD induced by this idealized approach in the following section.

### 3.3 Comparative analysis of WFD under different 3D alignments

**Fig 10:**
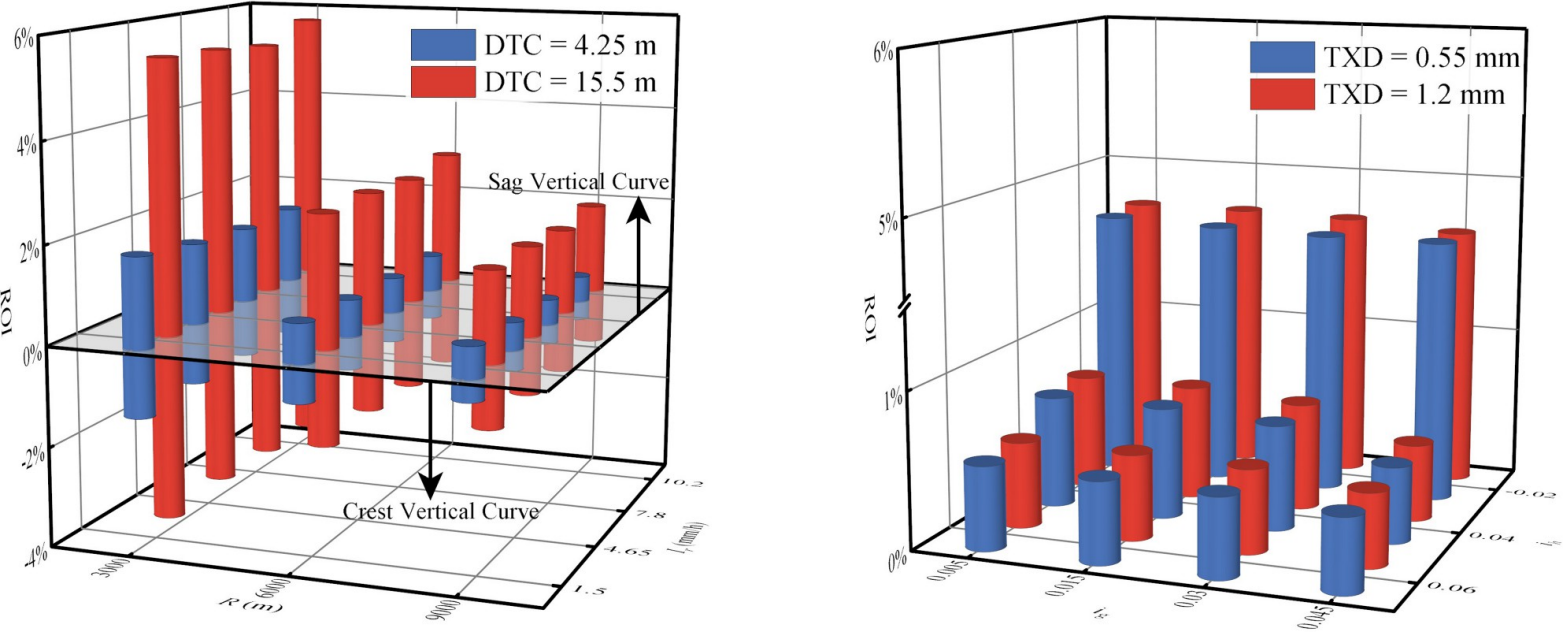
The difference in WFD between Type iii and Type i. (a) *i*_*h*_ = -0.02, *i*_*g*_ = 0.005, TXD = 0.55 mm. (b) Sag vertical curve, *I*_*r*_ = 7.8 mm/h, *R* = 3000 m, DTC = 15.5 m.

To visually assess the differences in WFD among Types ii and iii relative to Type i, we calculated the increase rate of WFD as defined in Eq (8). A positive ROI indicates that WFD for Types ii and iii surpasses that of Type i, while a negative ROI indicates lower WFD for Types ii and iii. As illustrated in Fig 10, with all other factors held constant, the sag vertical curves of Type ii show higher WFD compared to Type i, while the crest vertical curves display lower WFD. Further analysis reveals that greater DTC (*i*_*h*_ < 0), smaller superelevation values, and smaller vertical curve radii correlate with higher ROI. However, the grade has minimal impact on ROI. Numerical simulations suggest the ROI can exceed 5%, indicating that conventional models, which simplify the road’s longitudinal grade to a constant slope, may inaccurately predict WFD for Type ii to some extent. Additionally, TXD emerges as a significant factor, with smaller TXD generally associated with larger ROI, indicating that the predictive errors of conventional models may progressively increase with pavement polishing. Despite the relatively minor impact of rainfall intensity on ROI, its effect on WFD should not be considered negligible, as it similarly influences the WFD of Types ii and i. Consequently, the proposed model is capable of capturing the subtle variations in WFD induced by changes in the longitudinal slope of Type ii, thereby enhancing the accuracy of WFD predictions.


$$\text{ROI}=\frac{{\text{WFD}}_{\text{Type}\;(j+\text{i})}-{\text{WFD}}_{\text{Type}\;(j)}}{{\text{WFD}}_{\text{Type}\;(j)}}\times 100\%,\;j=\text{i},\text{ii}$$ (8)

**Fig 11:**
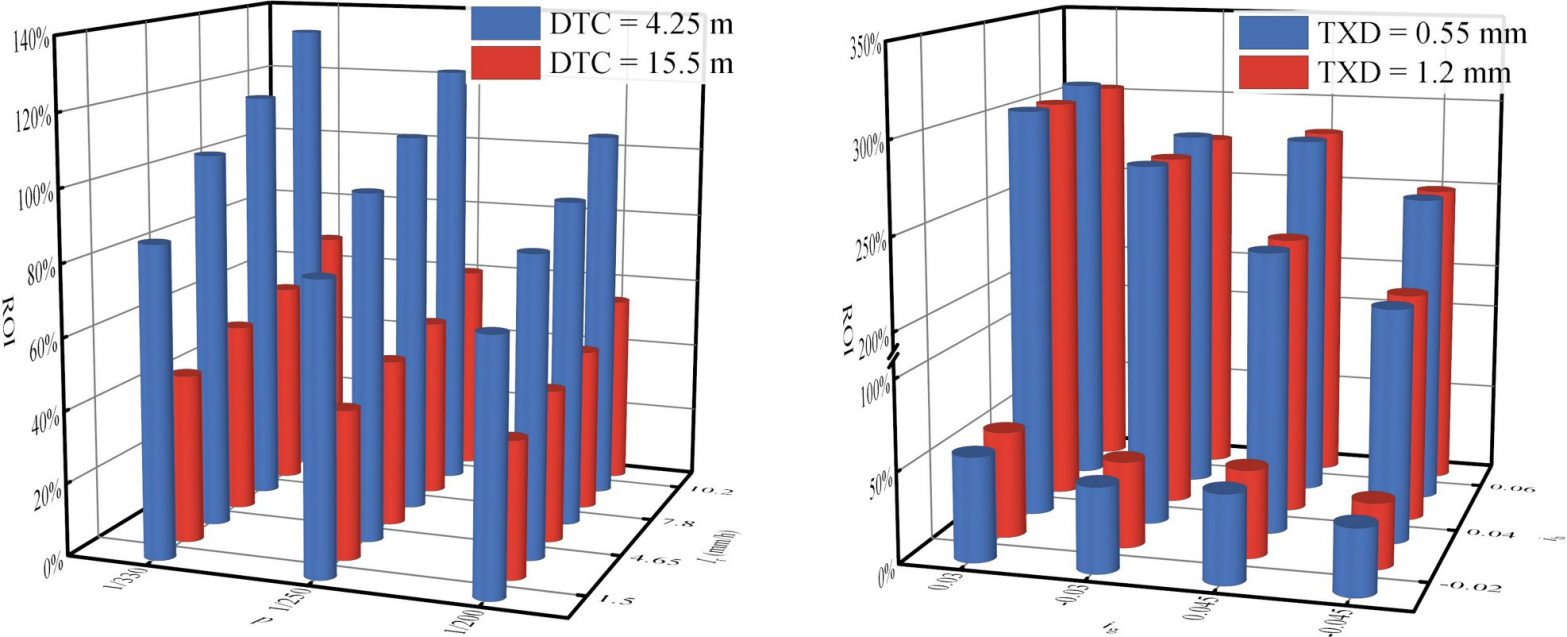
The disparity in WFD between Type iii and Type i. (a) *i*_*h*_ = -0.02, *i*_*g*_ = 0.03, TXD = 0.55 mm. (b) *I*_*r*_ = 7.8mm/h, *p* = 1/330, DTC = 15.5 m.

Fig 11 clearly illustrates the differences in WFD between Type iii and Type i. The results indicate that, with all other factors remaining constant, Type iii consistently exhibits a significantly higher WFD than Type i. Increases in DTC, superelevation, superelevation transition rate, and rainfall intensity, as well as reductions in longitudinal slope and TXD, contribute to an increase in the ROI. Within the range of numerical simulations, even the smallest observed ROI exceeds 35%. This phenomenon, often neglected in prior research, warrants attention due to its potential effect on driving safety. Historical studies have shown that injury and fatality rates on horizontal curves are over three times higher than on tangents, particularly in superelevation transitions [46]. Moreover, an increase in WFD reduces the friction coefficient between the pavement and the tire, further increasing the likelihood of vehicle skidding [20, 47]. Therefore, under rainy conditions, the thicker water film associated with Type iii could further heighten the risk of roadway collisions. Unfortunately, current research and practices aimed at reducing the impact of WFD on road safety primarily focus on speed control and roadside warning systems on circular curves [47–49], thereby overlooking the potential significance of Type iii in managing safety on rainy days. Thus, it is essential to enhance safety management measures for superelevation transitions.

The comparative analysis demonstrates that the DE-WFD model effectively captures variations in WFD caused by changes in longitudinal slope and superelevation, addressing the shortcomings of conventional models that fail to predict WFD across vertical curves and superelevation transitions. Notably, not all WFDs necessarily compromise road safety margins. This is because highway alignment design standards in various countries are generally based on wet pavement conditions. If the WFD does not meet this criterion of wetness, additional interventions during rainfall are not required by traffic managers. Therefore, determining the rainfall conditions under which various highway alignments meet this wet state is of significant practical value.

### 3.4 The rainfall conditions required to reach the critical WFD

The parameter of critical WFD describes wet pavement conditions. It represents the maximum WFD permissible under specified rainfall scenarios, where the tangential friction coefficient between the pavement and the tire decreases to a level consistent with the standard stopping sight distance. Kulakowski et al. [21] subjectively adopted a criterion defining the critical WFD as a 75% reduction in the pavement friction coefficient, a standard lacking theoretical justification. Consequently, this study uses the stopping sight distance, a significant aspect of road geometric design, as the basis for determining the critical WFD. This parameter, relevant under wet conditions, includes various elements such as driver reaction time, the tangential friction coefficient, and vehicle braking distance. It demonstrates how rainfall impacts road safety and clarifies the misconception that any presence of water film inherently compromises highway safety. See Eq (9) for details.

$$\text{SSD}=0.278Vt+0.039\frac{V^{2}}{f_{\text{T}}g}$$ (9)

with,

$$f_{\text{T}}=\eta f$$ (10)

where SSD represents the stopping sight distance, *V* denotes the vehicle’s operating speed, *t* is the driver’s braking reaction time, and *f* is the coefficient of friction between the pavement and the tire. The design value of the tangential friction coefficient, *f*_T_, is 0.29 at a design speed of 120 km/h [17]. The proportion of the longitudinal friction coefficient, denoted as *η*, typically ranges between 80% and 95% [18].

The pavement-tire friction coefficient, as described in Eq (10), is influenced by several factors including WFD, vehicle speed, pavement properties, and tire characteristics. This relationship is well-documented in the literature [47, 50]. Among the various models developed to predict the pavement-tire friction coefficient, the model proposed by Rose et al. [51] is particularly notable for its comprehensive consideration of these influencing factors.

$$f=\frac{1.901}{V^{0.72}}\left({\left(\frac{\text{TD}}{25.4}\right)}^{0.06}+\frac{3.556{\text{TXD}}^{0.05}}{{\left(\text{WFD}+2.5\right)}^{0.08}}\right),\;\text{ASTM}\;\text{Tire,}\;\text{221}\;{\text{kN/m}}^{\text{2}}$$ (11)

where TD denotes tread depth. For new passenger radial tires, the typical tread depth ranges from 8 to 10 mm, with a wear limit set at 1.6 mm.

The critical WFD is calculated using Eqs (9)-(11), with the result presented in Table 4.

**Table 4:** Critical WFD value.

Design speed (km/h)	*V* (km/h)	SSD (m)	*f*	Critical WFD (mm)
120	102	210	0.29	0.382

**Fig 12:**
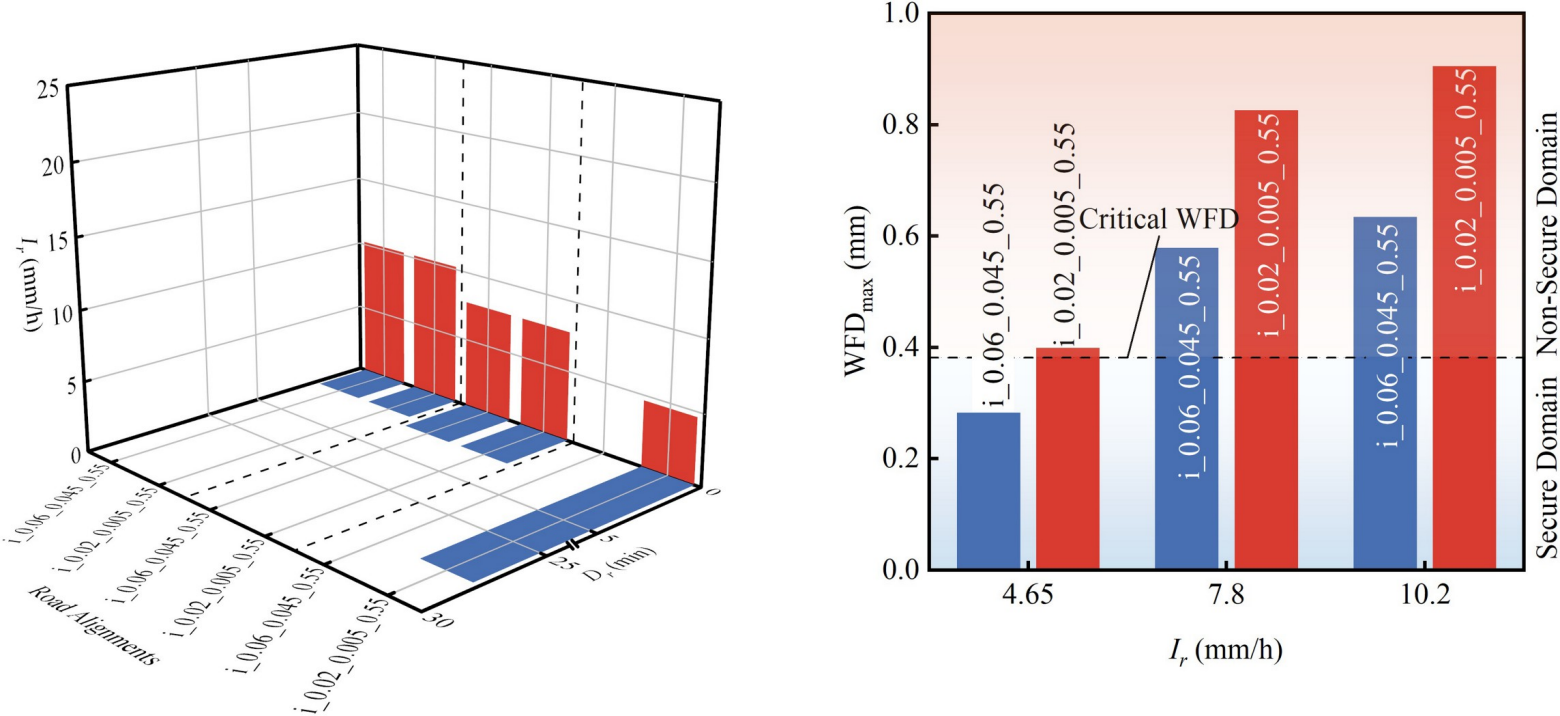
Rainfall conditions for reaching critical WFD and maximum WFD across different road alignments (Note: The values on the road alignments axis denote road type number_superelevation_grade_TXD). (a) Rainfall intensity and duration required to reach critical WFD. (b) Maximum WFD corresponding to the road segments and rainfall intensities described in (a).

Simulations based on Table 4 and the DE-WFD model were conducted to determine the rainfall intensity and duration (*D*_*r*_) required to achieve critical WFD for various road geometries. The selected results are illustrated in Fig 12. These findings indicate that critical WFD is primarily influenced by rainfall intensity and duration, with road geometry parameters having a minimal impact. For example, in the second comparison set shown in Fig 12, despite more than a 40% variation in maximum WFD between two road segments, the durations of rainfall required to achieve critical WFD are remarkably consistent, differing by only 1.2 seconds. Additionally, it was observed that an increase in rainfall intensity significantly reduces the time needed to reach critical WFD. Specifically, at intensities above 7.8 mm/h, critical WFD is achieved within three minutes. However, as rainfall intensity continues to increase, the rate of time reduction diminishes. Conversely, for rainfall intensities below 4.75 mm/h, as observed in the third group’s road segment with a longitudinal slope of 0.045 and a superelevation of 0.06, critical WFD is not reached, indicating that under lower rainfall intensities, this segment can provide safer driving conditions.

**Fig 13:**
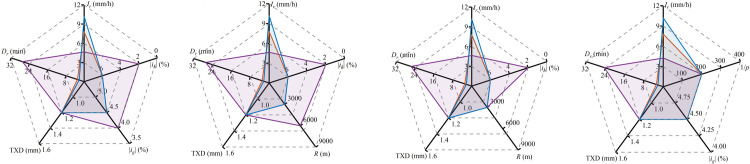
The rainfall conditions necessary to reach critical WFD. (a) Type i. (b) Type ii (Crest Vertical Curve). (c) Type ii (Sag Vertical Curve). (d) Type iii.

To enhance the practicality of the findings, Fig 13 illustrates the rainfall conditions associated with critical WFD. Each color boundary delineates the combined effects of rainfall intensity, highway alignments, and TXD on critical WFD. An outward expansion of these boundaries indicates that the critical WFD value has been exceeded, whereas an inward contraction suggests it has not been met. Fig 13 demonstrates that critical WFD, under various highway alignments, is influenced by both the intensity and duration of rainfall. For instance, on segments with longitudinal gradients and superelevations not exceeding ±4.5% and ±6%, respectively, a rainfall intensity exceeding 4.65 mm/h causes the WFD to reach the critical value within 27 minutes; at an intensity exceeding 7.8 mm/h, the threshold is achieved in as little as 2.95 minutes. It is important to note that the criteria for determining critical WFD depicted in Fig 13 are applicable under conditions of unstructured drainage and unobstructed flow. However, variations in drainage methods and configurations may alter the efficiency of water escape from the pavement [26], which could subsequently influence the rainfall intensity and duration necessary to reach critical WFD for a specific highway alignment and TXD.

In conclusion, accurately predicting WFD and identifying rainfall conditions that reach critical WFD remain challenging. These predictions are complicated by the diversity of road alignments and the transient nature of rainfall intensity [52]. Additionally, drainage methods and facility designs may influence WFD. Fortunately, contemporary technology has made these challenges more manageable. The implementation of inductive loops and sensors enables real-time monitoring of rainfall intensity, duration, and road surface drainage conditions. Roadway geometrics are readily obtainable from highway alignment design departments. Furthermore, several mathematical models have been developed to predict the maximum vehicle speed based on WFD, vehicular parameters, and highway alignments [47, 48]. Finally, with the integration of Internet of Things, combined positioning, and low-latency communication technologies, proactive control over safe speeds is achievable. Supported by these technologies, it is believed that safe access to expressways under rainy conditions can be robustly ensured.

## 4 Conclusion

Accurately predicting WFD under rainy conditions is essential for ensuring all-weather safety on expressways. This paper presents a DE-WFD model that simulates WFD across various road geometries under dispersed drainage conditions. The model is based on 3D road models constructed using BIM and a discrete phase-Eulerian wall film model developed with CFD. The DE-WFD model has demonstrated high accuracy and broad applicability through comparative analyses with conventional models and field test values. It confirms the technical feasibility of accurately replicating highway alignments with BIM and simulating WFD using the coupled discrete phase model and Eulerian wall film model, thereby significantly enhancing precision. The main conclusions are as follows:

- The DE-WFD model accurately predicts WFD under various highway alignments. In comparison to conventional models, including RRL, PAVDRN, and Gallaway, it can effectively capture the spatiotemporal variations in WFD across different road alignments. This capability addresses the limitations of classical models regarding alignment applicability and temporal domain, thereby improving the accuracy of WFD predictions.
- On road surfaces employing dispersed drainage, WFD is affected by rainfall conditions, road geometric characteristics, and the TXD of the pavement. WFD decreases with increases in the longitudinal slope, superelevation, and the rate of superelevation, while it increases with heightened rainfall intensity, duration, TXD, catchment width, and the radius of vertical curves. Additionally, in the superelevation transition segments, the maximum WFD is observed on the outer lane just before the zero cross slope section when descending, and the opposite is true when ascending.
- As rainfall intensity increases, the duration required to reach the critical WFD is reduced. Once the critical WFD threshold is reached, a significant adverse effect on vehicular safety is observed. Therefore, additional interventions, such as the issuance of warning messages or variable speed limit notifications, must be implemented by traffic management authorities to ensure road safety.
- Although the critical WFD serves as a threshold for evaluating its impact on highway safety, this paper does not explore the specific effects of WFD on safety. Research has shown that an increase in WFD significantly reduces the friction coefficient between the pavement and the tire [47], substantially impairing vehicle handling and braking performance. Therefore, a quantitative assessment of the extent to which WFD affects highway safety is necessary and warrants further in-depth investigation.

## Supporting information

S1 File Detailed data description. (XLSX)

## References

[1] Rasol M, Schmidt F, Ientile S, Adelaide L, Nedjar B, Kane M, et al. Progress and Monitoring Opportunities of Skid Resistance in Road Transport: A Critical Review and Road Sensors. Remote Sens. 2021;13: 3729. doi: 10.3390/rs13183729

[2] Jaroszweski D, McNamara T. The Influence of Rainfall on Road Accidents in Urban Areas: A Weather Radar Approach. Travel Behav Soc. 2014;1: 15–21. doi: 10.1016/j.tbs.2013.10.005

[3] Lee J, Chae J, Yoon T, Yang H. Traffic Accident Severity Analysis with Rain-Related Factors Using Structural Equation Modeling–a Case Study of Seoul City. Accid Anal Prev. 2018;112: 1–10. doi: 10.1016/j.aap.2017.12.013 29306084

[4] FHWA. Rain & Flooding. 2023 Feb 1 [cited 6 May 2024]. Available: https://ops.fhwa.dot.gov/weather/weather_events/rain_flooding.htm

[5] Jung S, Jang K, Yoon Y, Kang S. Contributing Factors to Vehicle to Vehicle Crash Frequency and Severity Under Rainfall. J Safety Res. 2014;50: 1–10. doi: 10.1016/j.jsr.2014.01.001 25142355

[6] Theofilatos A, Yannis G. A Review of the Effect of Traffic and Weather Characteristics on Road Safety. Accid Anal Prev. 2014;72: 244–256. doi: 10.1016/j.aap.2014.06.017 25086442

[7] Russam K, Ross NF. The Depth of Rain Water on Road Surfaces. London, UK: Road Research Laboratory, Minstry of Transport; 1968 p. 25.

[8] Gallaway BM, Rose JG, Hankins KD, Scott WW Jr, Schiller RE Jr. Influence of Water Depths on Friction Properties of Various Pavement Types. Austin, USA: Texas Highway Department in cooperation with U. S. Department of Transportation Federal Highway Administration; 1974.

[9] Han S, Xu J, Yan M, Gao S, Li X, Huang X, et al. Predicting the Water Film Depth: A Model Based on the Geometric Features of Road and Capacity of Drainage Facilities. Mosa AM, editor. PLOS ONE. 2021;16: e0252767. doi: 10.1371/journal.pone.0252767 34214083 PMC8253438

[10] Yang W, Tian B, Fang Y, Wu D, Zhou L, Cai J. Evaluation of Highway Hydroplaning Risk Based on 3D Laser Scanning and Water-Film Thickness Estimation. Int J Environ Res Public Health. 2022;19: 7699. doi: 10.3390/ijerph19137699 35805357 PMC9266007

[11] Tabatabai H, Aljuboori M. A Novel Concrete-Based Sensor for Detection of Ice and Water on Roads and Bridges. Sensors. 2017;17: 2912. doi: 10.3390/s17122912 29240710 PMC5750530

[12] Lubnow M, Dreier T, Schulz C, Endres T. Water-Film Thickness Imaging Based on Time-Multiplexed Near-Infrared Absorption with up to 500 Hz Repetition Rate. Appl Opt. 2023;62: 3169–3175. doi: 10.1364/AO.486206 37133165

[13] Ma Y, Geng Y, Chen X, Lu Y. Prediction for Asphalt Pavement Water Film Thickness Based on Artificial Neural Network. J Southeast Univ Engl Ed. 2017;33: 490–495.

[14] Luo W, Li L. Development of a New Analytical Water Film Depth (WFD) Prediction Model for Asphalt Pavement Drainage Evaluation. Constr Build Mater. 2019;218: 530–542. doi: 10.1016/j.conbuildmat.2019.05.142

[15] Jia X, Chen X, Huang P, Ma Q, Li S, Yan M. Influence of Geometric Alignment of Expressway Superelevation Transition Sections on Hydroplaning Speed of Minibuses. J Traffic Transp Eng. 2022;22: 140–147. doi: 10.19818/j.cnki.1671-1637.2022.04.010

[16] AASHTO. A Policy on Geometric Design of Highways and Streets. 7th ed. Washington, DC, USA: American Association of State Highway Transportation Officials; 2018.

[17] Ministry of Transport of the People’s Republic of China. Technical Standard of Highway Engineering. Beijing, China: China Communications Press; 2015.

[18] Lamm R, Psarianos B, Mailaender T. Highway Design and Traffic Safety Engineering Handbook. New York, NY, USA: McGraw-Hill Professional Publishing; 1999. Available: https://trid.trb.org/view/503709

[19] Besse J-P. Water Film Thickness Effects on the Friction Between Tire and Pavement. Pennsylvania State University. 1972.

[20] Do M-T, Cerezo V, Beautru Y, Kane M. Modeling of the Connection Road Surface Microtexture/Water Depth/Friction. Wear. 2013;302: 1426–1435. doi: 10.1016/j.wear.2013.01.031

[21] Kulakowski BT, Harwood DW. Effect of Water-Film Thickness on Tire-Pavement Friction. In: Surface Characteristics of Roadways: International Research and Technologie. Philadelphia, PA: American Society for Testing and Materials; 1990. pp. 50–60. Available: https://asmedigitalcollection.asme.org/astm-ebooks/book/1701/chapter/27835772/Effect-of-Water-Film-Thickness-on-Tire-Pavement

[22] Ding H, Zhang Y, Sun C, Yang Y, Wen C. Numerical Simulation of Supersonic Condensation Flows Using Eulerian-Lagrangian and Eulerian Wall Film Models. Energy. 2022;258: 124833. doi: 10.1016/j.energy.2022.124833

[23] Zhang S, Jacobs G, von Goeldel S, Vafaei S, König F. Prediction of Film Thickness in Starved Ehl Point Contacts Using Two-Phase Flow CFD Model. Tribol Int. 2023;178: 108103. doi: 10.1016/j.triboint.2022.108103

[24] Bhuiyan AA, Naser J. Development of 3D Transient Wall Filming Mechanism During Combustion by Coupling Eulerian-Lagrangian Approach and Particle-Wall Interaction Model. Appl Therm Eng. 2017;112: 911–923. doi: 10.1016/j.applthermaleng.2016.10.174

[25] Khoa ND, Kuga K, Inthavong K, Ito K. Coupled Eulerian Wall Film–Discrete Phase Model for Predicting Respiratory Droplet Generation During a Coughing Event. Phys Fluids. 2023;35: 112103. doi: 10.1063/5.0174014

[26] Aranda JÁ, Beneyto C, Sánchez-Juny M, Bladé E. Efficient Design of Road Drainage Systems. Water. 2021;13: 1661. doi: 10.3390/w13121661

[27] Mukherjee D. Highway Surface Drainage System & Problems of Water Logging in Road Section. Int J Eng Sci. 2014;3: 44–51.

[28] Pitzalis L, Livesu M, Cherchi G, Gobbetti E, Scateni R. Generalized adaptive refinement for grid-based hexahedral meshing. ACM Trans Graph. 2021;40: 1–13. doi: 10.1145/3478513.3480508

[29] Ansys. Ansys ICEM CFD Help Manual. Canonsburg, USA: ANSYS, Inc.; 2023. Available: https://ansyshelp.ansys.com/Views/Secured/corp/v231/en/icm_help/iedit_determinant.html

[30] Ansys. Ansys Fluent Theory Guide. Canonsburg, USA: ANSYS, Inc.; 2023.

[31] Dai R, Fu S, Yuan H. Study on the Wall Film Behavior and Droplet Catching Performance of a Micro Cyclone in Hydrogen Fuel Cells. Int J Hydrog Energy. 2024;61: 125–136. doi: 10.1016/j.ijhydene.2024.02.183

[32] Anzai H, Shindo Y, Kohata Y, Hasegawa M, Takana H, Matsunaga T, et al. Coupled Discrete Phase Model and Eulerian Wall Film Model for Numerical Simulation of Respiratory Droplet Generation During Coughing. Sci Rep. 2022;12: 14849. doi: 10.1038/s41598-022-18788-3 36050319 PMC9434508

[33] Van Dijk A, Bruijnzeel LA, Rosewell CJ. Rainfall Intensity–Kinetic Energy Relationships: A Critical Literature Appraisal. J Hydrol. 2002;261: 1–23. doi: 10.1016/S0022-1694(02)00020-3

[34] Angulo-Martínez M, Beguería S, Kyselỳ J. Use of Disdrometer Data to Evaluate the Relationship of Rainfall Kinetic Energy and Intensity (KE-I). Sci Total Environ. 2016;568: 83–94. doi: 10.1016/j.scitotenv.2016.05.223 27288763

[35] Torres DS, Salles C, Creutin JD, Delrieu G. Quantification of Soil Detachment by Raindrop Impact: Performance of Classical Formulae of Kinetic Energy in Mediterranean Storms. IAHS Publ. 1992;210: 115–124.

[36] Cristiano E, ten Veldhuis M-C, van de Giesen N. Spatial and Temporal Variability of Rainfall and Their Effects on Hydrological Response in Urban Areas–a Review. Hydrol Earth Syst Sci. 2017;21: 3859–3878. doi: 10.5194/hess-21-3859-2017

[37] Plati C, Pomoni M. Impact of Traffic Volume on Pavement Macrotexture and Skid Resistance Long-Term Performance. Transp Res Rec J Transp Res Board. 2019;2673: 314–322. doi: 10.1177/0361198118821343

[38] Lu C, Zhang Z, Qiang Y, Zhao F, Wang D. A Hydrophobic and Sustainable Anti-Icing Sand Fog Seal for Asphalt Pavement: Its Preparation and Characterization. Constr Build Mater. 2023;401: 132918. doi: 10.1016/j.conbuildmat.2023.132918

[39] Zhang Z, Lu C, Qiang Y, Guo Y, Dong D, Zhao F. Preparation and Properties Analysis of Antifreezes and Fogseal. J Jilin Univ Eng Technol Ed. 2023;53: 3492–3500. doi: 10.13229/j.cnki.jdxbgxb.20220089

[40] Vakamalla TR, Mangadoddy N. Numerical Simulation of Industrial Hydrocyclones Performance: Role of Turbulence Modelling. Sep Purif Technol. 2017;176: 23–39. doi: 10.1016/j.seppur.2016.11.049

[41] Wikipedia. Feeler Gauge. 2024 Feb 27 [cited 15 Mar 2024]. Available: https://en.wikipedia.org/w/index.php?title=Feeler_gauge&oldid=1210514639

[42] Yang D, Zhou Q. Experimental Study of Relationship Between Voidage and Permeability Coefficient of Asphalt Mixture. J Chongqing Jiaotong Univ Nat Sci. 2019;38: 51–56.

[43] Chen S, Adhikari S, You Z. Relationship of Coefficient of Permeability, Porosity, and Air Voids in Fine-Graded Hma. J Mater Civ Eng. 2019;31: 4018359. doi: 10.1061/(ASCE)MT.1943-5533.0002573

[44] Anderson DA, Huebner RS, Reed JR, Warner JC, Henry JJ. Improved Surface Drainage of Pavements. 1998 Jun. Available: https://trid.trb.org/view/500507

[45] Zhao J, Guo W, Jia X, Chen X. Numerical simulation and law analysis of water accumulation distribution at superelevation transition section of multilane expressway. J Traffic Transp Eng. 2022;22: 187–196. doi: 10.19818/j.cnki.1671-1637.2022.02.014

[46] Gooch JP, Gayah VV, Donnell ET. Safety Performance Functions for Horizontal Curves and Tangents on Two Lane, Two Way Rural Roads. Accid Anal Prev. 2018;120. doi: 10.1016/j.aap.2018.07.030 30077907

[47] Peng J, Chu L, Wang T, Fwa TF. Analysis of Vehicle Skidding Potential on Horizontal Curves. Accid Anal Prev. 2021;152: 105960. doi: 10.1016/j.aap.2020.105960 33540346

[48] Yan M, Xu J, Han S, Xin T, Wang O, Yi Z, et al. Permitted Speed Decision of Single-Unit Trucks with Emergency Braking Maneuver on Horizontal Curves Under Rainy Weather. PLOS ONE. 2021;16: e0261975. doi: 10.1371/journal.pone.0261975 34969049 PMC8717978

[49] Chen X, Wang H. Analysis and Mitigation of Hydroplaning Risk Considering Spatial-Temporal Water Condition on the Pavement Surface. Int J Pavement Eng. 2023;24: 2036988. doi: 10.1080/10298436.2022.2036988

[50] Do M, Cerezo V, Beautru Y, Kane M. Influence of Thin Water Film on Skid Resistance. J Traffic Transp Eng. 2014;2: 36–44.

[51] Rose JG, Gallaway BM. Water Depth Influence on Pavement Friction. Transp Eng J ASCE. 1977;103: 491–506. doi: 10.1061/TPEJAN.0000648

[52] Ressel W, Wolff A, Alber S, Rucker I. Modelling and Simulation of Pavement Drainage. Int J Pavement Eng. 2019;20: 801–810. doi: 10.1080/10298436.2017.1347437

